# When Do Orthopaedic Oncologists Consider the Implantation of Expandable Prostheses in Bone Sarcoma Patients?

**DOI:** 10.1155/2018/3504075

**Published:** 2018-02-25

**Authors:** Magdalena M. Gilg, Christine Wibmer, Marko Bergovec, Robert J. Grimer, Andreas Leithner

**Affiliations:** ^1^Department of Orthopaedic Surgery and Traumatology, Medical University of Graz, Graz, Austria; ^2^Royal Orthopaedic Hospital NHS Foundation Trust, Birmingham, UK

## Abstract

**Introduction:**

Indications discussed for the implantation of expandable prostheses in bone sarcoma patients are unclear. This survey aimed to analyse common practice with this implant type in orthopaedic oncology.

**Methods:**

A web-based survey was sent to 98 orthopaedic oncology surgeons. Factors reported in literature to influence the decision on the implantation of a growing prosthesis were covered in individual questions and three case scenarios.

**Results:**

The completion rate of the survey was 45% (*n* = 44). Twenty-seven of 44 surgeons (61%) had implanted between 1 and 15 expandable prostheses within three years. The minimum median patient age was 6.5 years, and 3–5 cm of predicted growth deficit was the minimum before implanting a growing prosthesis. One-third of surgeons do not use growth calculation methods. Two out of three surgeons would rather not implant a growing prosthesis in children with metastatic disease.

**Conclusions:**

Our survey confirmed the literature with 3-4 cm as the minimum estimated growth deficit. The minimum age for the implantation of a growing prosthesis is approx. 6.6 years, and therefore the patients are younger than those reported in previous publications. One-quarter of orthopaedic surgeons do not use growing prostheses at all. It remains unclear whether growing prostheses are indicated in patients with metastatic disease.

## 1. Introduction

Paediatric bone sarcoma frequently arises in the metadiaphyseal regions of the distal femur or the proximal tibia. Wide resection can include the growth plate, and there will be a leg-length discrepancy by skeletal maturity. In the past, amputations in the very young patients or multiple revision surgeries were performed to address the leg-length discrepancy [1].

In 1976, the first expandable prostheses (synonyms: extendible or growing prostheses) were introduced, allowing minimally invasive lengthening via a small skin incision [2]. Still, general anaesthetics and surgical interventions were required, which ultimately increased the risk of infection and the loss of prosthesis [3], so that noninvasively expandable prostheses are now considered to be the gold standard in paediatric limb-salvage surgery [4]. Different types of noninvasive lengthening mechanisms are currently available and can be applied in an outpatient setting [1, 2, 5, 6].

To implant a growing prosthesis, at least 3-4 cm of growth has to be expected for the child until skeletal maturity [7–9]. Furthermore, expandable prostheses require a minimal resection length between 123 and 170 mm depending on the prosthesis type and a sufficient bone diameter, limiting its use in very young children [6, 10]. There is no consensus on the minimum age for the implantation of a growing prosthesis [4, 6, 8, 10, 11].

Our survey aimed to clarify the indications for implantation of a growing prosthesis in bone sarcoma patients by conducting a survey among experts in orthopaedic oncology. Furthermore, we aimed to identify alternative methods other than expandable prostheses to compensate for limb-length inequality.

## 2. Materials and Methods

A ten-minute web-based survey (Question Pro©) was distributed via email to 98 active orthopaedic surgeons of the European Musculo-Skeletal Oncology Society (EMSOS) (Supplementary Materials (available here)). Nonorthopaedic members were not invited to participate in the survey. Participants who stated via email that they are retired or do not operate on children were excluded from the survey (2 surgeons). Forty-four invited orthopaedic surgeons from thirteen European countries responded to the survey (participation rate 45%), and the completion rate was 93%.

The questionnaire consisted of 15 items on 3 pages including case-specific questions. The first three survey questions asked about participants' personal experience with expandable prostheses, including years in practice, previous experience in orthopaedic oncology, and experience with the implantation of growing prostheses over the last three years. Questions 4–10 were based on relevant factors reported in literature (minimum age, minimum predicted growth, metastatic disease, and “dummy prostheses,” i.e., a growing prosthesis implanted without a motor immediately after wide resection, with implantation of the motor at a later stage). Questions 11 and 12 asked for other methods to maintain limb-length equality including epiphysiodesis. Finally, to check consistency, there were three case scenarios based on osteosarcoma patients aged 6.5, 8, and 10.5 years. For all three case scenarios, bone sarcomas were located in the distal femur and tumour extent was depicted. Total femur length as well as required minimal resection lengths of different types of growing prostheses was provided for each case. In addition to multiple choice answers, survey participants could provide information on their own surgical technique in a separate comment field. None of the cases involved skip metastasis, metastatic disease, intra-articular tumour infiltration, or pathological fracture.

The survey was constructed according to the “Checklist for Reporting Results of Internet E-Surveys (CHERRIES)” [12]. Before the survey was distributed, three specialists in orthopaedic oncology tested it for usability and technical functionality as well as comprehensibility. The survey was accessible only via email invitation with password protection, and unique visitors were identified by IP address. Survey visitors who did not start the questionnaire were excluded. All surveys were completed within a normal time frame. Duplicate database entries having the same user ID were eliminated before analysis, and the most complete entry was kept for analysis.

Statistical analysis was conducted with Microsoft Excel (Excel Version 2010). Categorical variables are presented as absolute and relative frequencies and numerical variables as means and ranges.

## 3. Results

Regarding personal experience, about one-half of the participants have been in practice for more than 20 years and two out of three orthopaedic surgeons dedicated more than 50% of their working time to musculoskeletal oncology. Twenty-seven of 44 surgeons (61%) had implanted between 1 and 15 expandable prostheses within the last three years, whereas about 30% (*n* = 13) of survey participants had not implanted any (Table 1).

**Figure 1:**
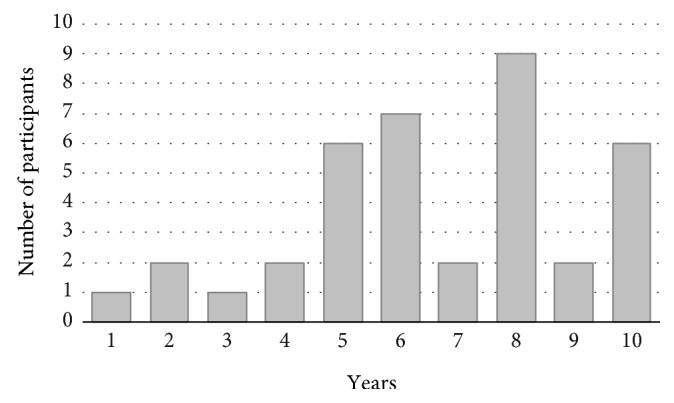
Minimum ages at the time of implantation.

**Figure 2:**
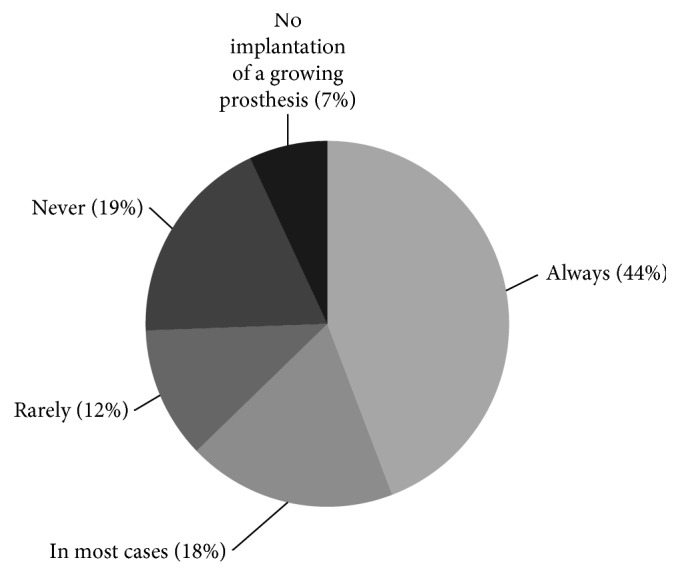
Growth prediction methods used by the survey participants (multiple answers possible).

The answers concerning factors influencing the decision for or against a growing prosthesis revealed a median minimum age of 6.5 years (range, 1–10) (Figure 1). The minimum predicted growth was given as 3–5 cm by a majority of participants (*n* = 26, 59%). To calculate the growth potential, one specific method is used by 45% (18/40), multiple methods by 20% (8/40), and none by 35% (14/40) of surgeons (Figure 2). In detail, Paley's multiplier method [13] is most frequently applied (9/40, 23%), followed by bone age estimation (8/40, 20%) and growth charts (6/40, 15%). About one-third of surgeons (12/39, 31%) would consider the implantation of an expandable prosthesis despite the presence of metastatic disease. The use of dummy prostheses is supported by 21% (8/38) of surgeons.

As alternative surgical option, about one-half of orthopaedic surgeons (20/43, 47%) would lengthen by callus distraction, either with intramedullary nailing devices or with Ilizarov technique. Additionally, 40% (17/42) of participants often or always consider epiphysiodesis as an option to guide growth.

**Figure 3:**
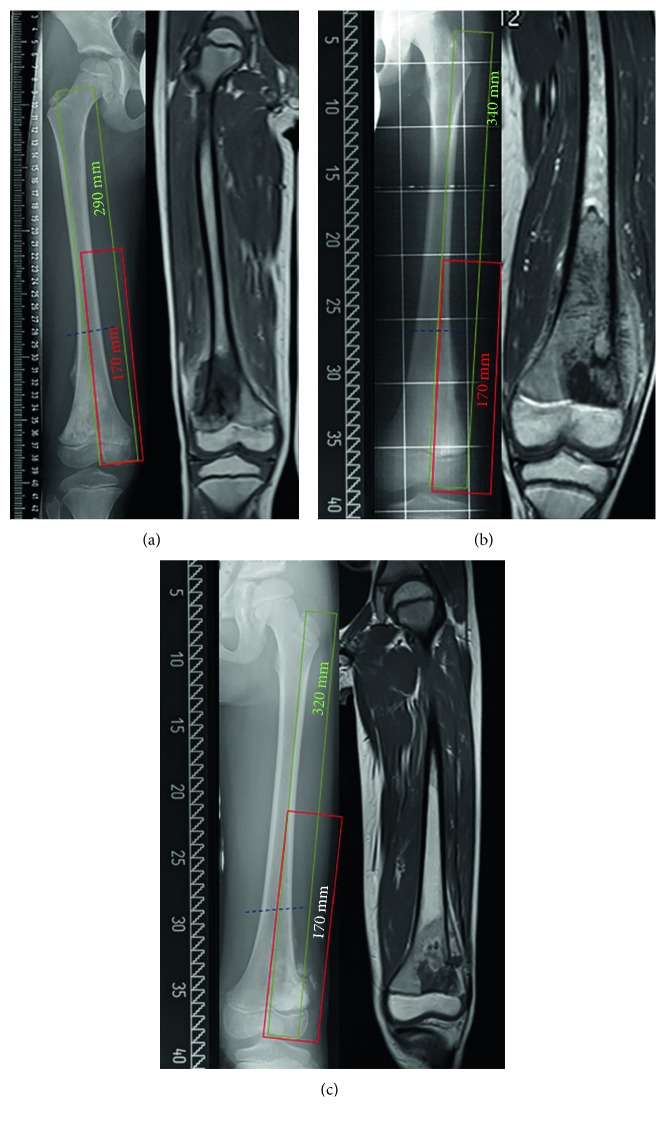
X-ray and MRI showing three case scenarios including specific answers for treatment options. None of the cases involved skip metastasis, metastatic disease, intra-articular tumour infiltration, or pathological fracture. (a) Male, 6.5 years at initial diagnosis, osteosarcoma of distal femur, and femur length (greater trochanter to eminentia intercondylaris) is 290 mm. (b) Female, 8.0 years at initial diagnosis, osteosarcoma of distal femur, and femur length (greater trochanter to eminentia intercondylaris) is 340 mm. (c) Male, 10.5 years at initial diagnosis, osteosarcoma of distal femur, and femur length (greater trochanter to eminentia intercondylaris) is 320 mm.

All three case scenarios were based on patients prior to the pubertal growth spurt (Figure 3). In line with the answers given in the survey questions, the implantation rate of a growing prosthesis increased from 53% (*n* = 23/43) for Case A to 76% (*n* = 32/42) and 83% (*n* = 34/41) for Cases B and C. Amputation was not considered by any of the respondents in any of the three case scenarios. Approximately one-quarter of surgeons (26%, *n* = 11/43) would use other surgical options for Case A, 14% (*n* = 6/42) for Case B, and 12% (*n* = 5/41) for Case C (Table 2).

## 4. Discussion

The use of expandable prostheses is one possible reconstruction method for children in whom limb-salvage surgery is feasible and sarcoma resection will result in significant leg-length discrepancy, and the literature fails to define the circumstances under which these prostheses are indicated. This survey aimed to identify expert opinions and consensus factors.

In our study population of experienced orthopaedic oncologists, 3-4 cm was confirmed as the minimum of estimated growth deficit prior to the implantation of an expandable device. The median minimum age for this surgical procedure was 6.5 (range 2–10) years. Apart from that, answers for specific factors were quite heterogeneous for the remaining questions. Demographics of survey participants, including years in practice and percentage of time dedicated to orthopaedic oncology, were representative of an expert population. It is unclear why one-third of participating surgeons do not consider the implantation of a growing prosthesis. Apart from individual surgical preferences, nonmedical reasons like the availability of implants in some countries and other socioeconomic reasons might influence the use of expandable prostheses.

We looked at factors influencing the surgical indication for a growing prosthesis that have been described in the literature, including age, expected growth deficit, growth prediction methods, metastatic disease, and alternative treatment options to compensate for leg-length discrepancy [1, 6–10]. Age has been described as a limiting factor since enough bone stock has to be available, and the minimum resection length varies between 123 and 170 mm for the implantation of a growing prosthesis. Furthermore, the younger the patient at first implantation, the more revision surgeries will be needed to overcome the maximum expansion capacities of implants. Our survey results depict a younger patient age than reported previously. Schinhan et al. [9] and Weisstein et al. [14] recommend growing prostheses in patients older than 8 years, whereas Yoshida et al. [15] suggest 10 years and older in their review articles. Staals et al. [8] reported using expandable prostheses in the 7- to 10-year age group. Apart from that, there are only descriptive data from single-centre analyses, with minimum ages between 5.5 and 8.2 years [11, 16, 17]. These studies did not include an outcome analysis correlating age at implantation with the amount of lengthening and the number of complications, and age recommendations do not differentiate between genders. As to age limits, there are neither validated recommendations nor an expert consensus.

It is important to predict growth accurately since 3-4 cm of remaining growth is seen as an indication for a growing prosthesis [7–9]. This calculation can be quite challenging, especially in children who undergo polychemotherapy, and to date there is no clear preference for a particular method [18]. In our survey, surgeons favoured Paley's multiplier method, bone age estimation, and growth charts. Interestingly, about one-third of survey participants do not conduct growth estimation prior to the implantation of an expandable implant. Baumgart and Lenze [7] emphasized the importance of a reliable method to calculate for growth potential and recommend Paley's multiplier method. Grimer et al. [1] use growth charts and bone age estimation with hand radiographs to obtain prospective data. Neither literature nor our survey results provide conclusive information as to which method is best applicable to this particular patient group.

Opinions as to whether expandable implants should be considered in patients with metastatic disease were divisive, though a majority would rather not use them with metastatic disease. The literature offers only minimal information, apart from Schinhan et al. [9], who see metastases at the time of implantation as a contraindication, but it must be taken into account that only 10–20% of patients have macroscopic evidence of metastatic disease, whereas 80–90% of patients with osteosarcoma are assumed to have micrometastatic disease at initial diagnosis [19].

Since this made it difficult to decide which patients would have a favourable prognosis at the time of implantation, some centres began to use dummy prostheses. The costly motor is implanted only later since response to chemotherapy cannot be predicted before surgery. It must also be considered here that chemotherapy reduces the growth velocity and most relapses occur within 2-3 years following initial diagnosis. There is no peer-reviewed literature on this practice, but our survey results showed that a considerable number of surgeons do use dummy prostheses.

The use of growing prostheses is only one possibility to compensate for leg-length discrepancy. In the case scenarios, we could see that the younger the patients were, the more cautious the participants were in opting for an expandable prosthesis. Instead, participants proposed biological reconstruction, temporary spacer, or callus distraction techniques. Our survey does not explain why one-third of surgeons do not consider expandable implants. In any case, alternative treatment option for paediatric sarcoma patients has to be discussed, since the currently available noninvasive expandable prostheses are still associated with complications, such as prosthetic joint infection or mechanical failure [4, 8]. Endoprosthetic devices are, however, in a phase of intensive and continuing development, and long-term outcome data are awaited with great interest.

## 5. Conclusions

Extendable implants were constructed to compensate for the limb-length discrepancy following metaepiphyseal sarcoma resection. At least 3-4 cm of leg-length discrepancy should be expected before a growing prosthesis is considered. Otherwise, there is no consensus on surgical indications among orthopaedic oncologists. Our survey showed that the opinions of experts in orthopaedic oncology from a wide geographic range are quite divergent that it would be a matter of some priority to work toward a consensus on the use of expandable prostheses in paediatric bone sarcoma patients.

## Figures and Tables

**Table 1:** Participants' characteristics.

*Years in practice*	*n*	%
0–5	2	5
6–10	3	7
11–15	14	32
16–20	3	7
>20	22	50
*Percentage of practice dedicated to musculoskeletal oncology*	*n*	%
0–25	3	7
26–50	7	16
51–75	8	19
76–100	25	58
*Number of growing prostheses implanted over the last 3 years*	*n*	%
0	13	30
1–5	14	32
6–15	13	30
16–25	1	2
26–35	1	2
>35	2	5

**Table 2:** Case scenarios A–C and possible alternative treatment options.

Case scenario	Expandable prosthesis	Amputation	Rotationplasty	Conventional tumour prosthesis	Other techniques	
Case A	53%	0%	16%	5%	26%	Allograft reconstruction (4), spacer techniques (2), callus distraction by intramedullary nailing devices (2), Canadell's epiphyseal distraction (1), “Pamplona technique” (1), decision according to family (1)
Case B	76%	0%	7%	3%	14%	Allograft reconstruction (3), spacer techniques (1), callus distraction by intramedullary nailing devices (1), Canadell's epiphyseal distraction (1)
Case C	83%	0%	0%	5%	12%	Allograft reconstruction (3), intraepiphyseal distraction and allograft reconstruction (1), Canadell's epiphyseal distraction (1)

## Supplementary Materials

Supplementary Materials Survey questions.
