# Supplementary material for: When Do Orthopaedic Oncologists Consider the Implantation of Expandable Prostheses in Bone Sarcoma Patients?
Source: Sarcoma. 2018 Feb 25;2018:3504075. doi: 10.1155/2018/3504075 (PMC5845529; doi:10.1155/2018/3504075)
Supplement: Supplementary Materials — Survey questions. [file 3504075.f1.pdf]

## Survey: EMSOS

Indications for the implantation of growing prosthesis in children with malignant bone sarcoma of the distal femur-
A survey among members of the European Musculo-Skeletal Oncology Society

**Dear colleagues,**
**You are invited to participate in our survey. Approximately 90 members of the EMSOS will be asked to complete a survey that asks questions about growing prostheses in children with bone sarcoma. It will take approximately 5 minutes to complete the questionnaire.**

**Your survey responses will be strictly confidential and data from this research will be reported only in the aggregate. Your information will be coded and will remain confidential. If you have questions you may contact M. Gilg (magdalena.gilg@medunigraz.at).**

**Thank you very much for your time and support. Please start with the survey now by clicking on the Continue button below.**

---

**How many years have you been in practice ?**

- ○ 0-5
- ○ 6-10
- ○ 11-15
- ○ 16-20
- ○ >20

---

**What percentage of your practice is dedicated to musculoskeletal oncology ?**

- ○ 0%-25%
- ○ 26%-50%
- ○ 51%-75%
- ○ 76%-100%

---

**How many growing prostheses have you implanted over the <u>last three years</u> ?**

- ○ 0
- ○ 1- 5
- ○ 6-15
- ○ 16-25
- ○ 26-35
- ○ > 35

---

**What do you consider the minimum age for the implantation of a growing prosthesis in a child ?**

- ☐ no implantation of a growing prosthesis in our center
- ☐ Age (years) ________

---

**What is the minimum amount of predicted growth until you implant a growing prosthesis in a child ?**

- ○ 2-3 cm
- ○ 3-4 cm
- ○ 4-5 cm
- ○ 5-6 cm
- ○ no implantation of a growing prosthesis in our center
- ○ other (in cm) [ ]

---

**Do you consider implanting a "Dummy" prosthesis (growing prosthesis without motor ) immediately after wide resection in children with bone sarcomas and implantation of the motor at a later stage (e.g. after 1 year follow-up) ?**

- ○ in most cases
- ○ rarely
- ○ never
- ○ I am not sure
- ○ no implantation of a growing prosthesis in our center

---

**Do you consider implanting a growing prosthesis in children with metastatic disease ?**

- ○ always
- ○ in most cases
- ○ rarely
- ○ never
- ○ I am not sure
- ○ no implantation of growing prosthesis in our center

---

**Do you use special methods to calculate remaining growth when planning a growing prosthesis?**

- ○ always
- ○ in most cases
- ○ rarely
- ○ never
- ○ I am not sure
- ○ no implantation of growing prosthesis in our center

---

**Which type(s) of method(s) is(are) used in your center to calculate remaining growth in pediatric bone sarcoma patients (if not using any, please type in "no method used")?**

[ ]

---

**Do you use epiphyseodesis to guide growth in children with leg length discrepancy?**

- ○ always
- ○ in most cases
- ○ rarely

- never
- I am not sure

**Do you use <u>other</u> methods of leg lengthening?**

- No
- Yes, we use :

**CASE A)**

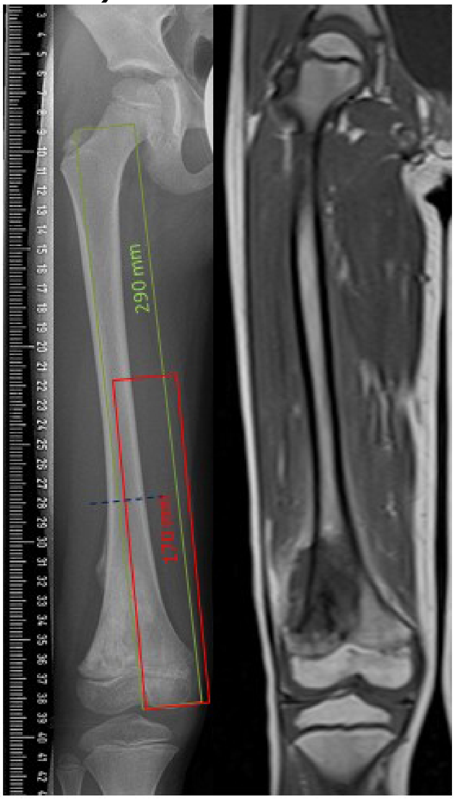


**male, 6.5 years at initial diagnosis**
**osteosarcoma of distal femur**
**no skip metastasis, no metastatic disease, no intraarticular tumour infiltration, no pathological fracture**
**femur length (greater trochanter to eminentia intercondylaris): 290 mm**
**proximal tumor border = - - -**

| | extension (mm) | minimum resection length (mm) |
|---|---|---|
| REPIPHYSIS® | 35 | 123-147 |
| | 60 | 148-173 |
| | 85 | 174-199 |
| | 110 | > 200 |
| MUTARS® Xpand | 50 | 170 |
| | 75 | 195 |
| | 100 | 220 |
| Non-invasive JTS | 50 | 170 |
| Implants (Stanmore) | 70 | 190 |
| | 90 | 210 |

**Which treatment would you recommend?**

- Implantation of a growing prosthesis

- Amputation
- Rotationplasty
- Implantation of a conventional tumor prosthesis
- Other

---

**CASE B)**

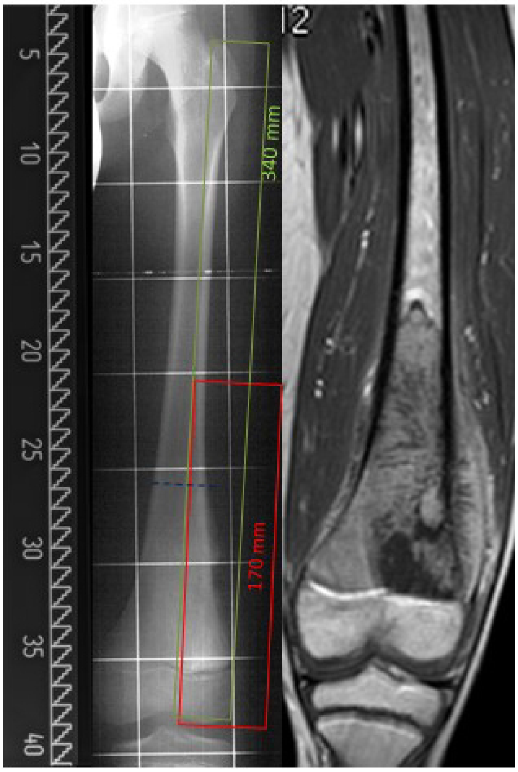


**female, 8.0 years at initial diagnosis**
**osteosarcoma of distal femur**
**no skip metastasis, no metastatic disease, no intraarticular tumour infiltration, no pathological fracture**
**femur length (greater trochanter to eminentia intercondylaris): 340 mm**
**proximal tumor border= - - -**

| | extension (mm) | minimum resection length (mm) |
|---|---|---|
| REPIPHYSIS® | 35 | 123-147 |
| | 60 | 148-173 |
| | 85 | 174-199 |
| | 110 | > 200 |
| MUTARS® Xpand | 50 | 170 |
| | 75 | 195 |
| | 100 | 220 |
| Non-invasive JTS Implants (Stanmore) | 50 | 170 |
| | 70 | 190 |
| | 90 | 210 |

**Which treatment would you recommend?**

- Implantation of a growing prosthesis
- Amputation
- Rotationplasty
- Implantation of a conventional tumor prosthesis
- Other

**CASE C)**

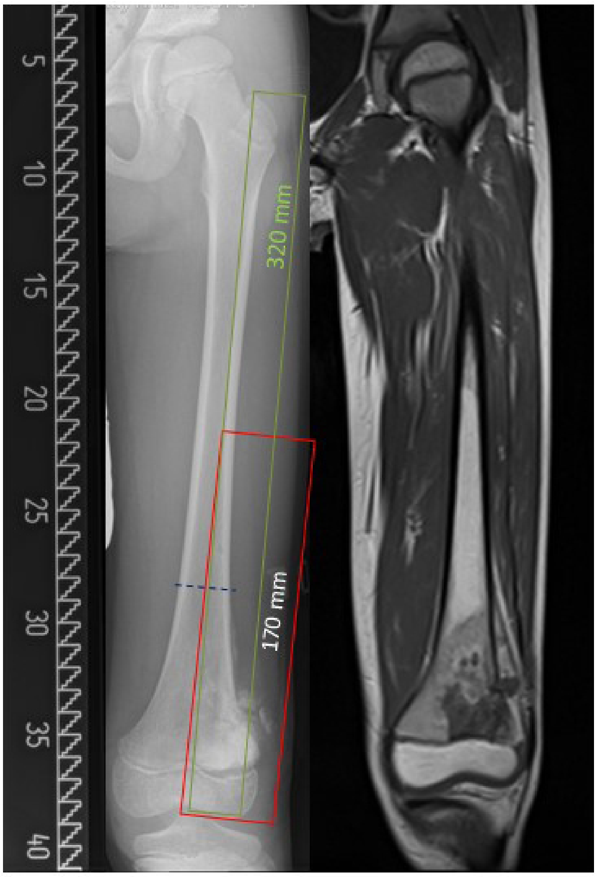


**male, 10.5 years at initial diagnosis**
**osteosarcoma of distal femur,**
**no skip metastasis, no metastatic disease, no intraarticular tumour infiltration, no pathological fracture**
**femur length (greater trochanter to eminentia intercondylaris): 320 mm**
**proximal tumor border= - - -**

| | extension (mm) | minimum resection length (mm) |
|---|---|---|
| REPIPHYSIS® | 35 | 123-147 |
| | 60 | 148-173 |
| | 85 | 174-199 |
| | 110 | > 200 |
| MUTARS® Xpand | 50 | 170 |
| | 75 | 195 |
| | 100 | 220 |
| Non-invasive JTS Implants (Stanmore) | 50 | 170 |
| | 70 | 190 |
| | 90 | 210 |

**Which treatment would you recommend?**

- ○ Implantation of a growing prosthesis
- ○ Amputation
- ○ Rotationplasty
- ○ Implantation of a conventional tumor prothesis
- ○ Other

**Please use this space for comments or suggestions on the survey. Thank you!**

Share This Survey:

Online Survey Software Powered by QuestionPro
